# Promoting Osteogenic Differentiation of Human Adipose-Derived Stem Cells by Altering the Expression of Exosomal miRNA

**DOI:** 10.1155/2019/1351860

**Published:** 2019-07-01

**Authors:** Shude Yang, Shu Guo, Shuang Tong, Xu Sun

**Affiliations:** Department of Plastic Surgery, The First Hospital of China Medical University, No. 155, Nanjing North Street, Heping District, Shenyang, 110002 Liaoning Province, China

## Abstract

Human adipose-derived stem cells (ADSCs) can release exosomes; however, their specific functions remain elusive. In this study, we verified that exosomes derived from osteogenically differentiated ADSCs can promote osteogenic differentiation of ADSCs. Furthermore, in order to investigate the importance of exosomal microRNAs (miRNAs) in osteogenic differentiation of ADSCs, we used microarray assays to analyze the expression profiles of exosomal miRNAs derived from undifferentiated as well as osteogenically differentiated ADSCs; 201 miRNAs were upregulated and 33 miRNAs were downregulated between the two types of exosomes. Additionally, bioinformatic analyses, which included gene ontology analyses, pathway analysis, and miRNA-mRNA-network investigations, were performed. The results of these analyses revealed that the differentially expressed exosomal miRNAs participate in multiple biological processes, such as gene expression, synthesis of biomolecules, cell development, differentiation, and signal transduction, among others. Moreover, we found that these differentially expressed exosomal miRNAs connect osteogenic differentiation to processes such as axon guidance, MAPK signaling, and Wnt signaling. To the best of our knowledge, this is the first study to identify and characterize exosomal miRNAs derived from osteogenically differentiated ADSCs. This study confirms that alterations in the expression of exosomal miRNAs can promote osteogenic differentiation of ADSCs, which also provides the foundation for further research on the regulatory functions of exosomal miRNAs in the context of ADSC osteogenesis.

## 1. Introduction

Effective reconstruction of craniomaxillofacial bone defects caused by trauma, tumor resection, or congenital malformation is a major problem in orthopedic surgery. In most cases, bones can regenerate and heal themselves [1]. However, this ability is lost when the area of the bone defect exceeds a critical size. In clinical practice, autologous and allogenic bone grafts can be a “gold standard” in bone defect treatment, even they have certain limitations such as chronic pain, poor cosmesis, nonunion, and infection [2, 3]. Over the last decades, mesenchymal stem cells (MSCs) have attracted extensive attention in the field of bone regeneration [4–7].

MSCs are a population of nonhematopoietic adult stem cells that have the property of self-renewal and can differentiate into multiple lineages [8–10]. They were initially found in the bone marrow [11] but can also be found in other tissues, such as adipose, periosteum, muscle, placenta, and trabecular bone [12]. Among these, adipose-derived stem cells (ADSCs) are a type of mesenchymal stem cell isolated from adipose tissue, which has the advantages of abundant storage *in vivo*, easy acquisition, and expansion [13–15]. Recently, a number of studies have confirmed that ADSCs possess the ability to differentiate into adipocytes, osteoblasts, and chondrocytes [16–18], suggesting that a broader source of stem cells is available for application in tissue engineering. ADSCs have already been used in bone regeneration [19, 20].

With the in-depth researches of MSCs, an increasing number of studies have indicated that the therapeutic effects of MSCs can be attributed not only to their differentiation capacity but also to their paracrine action [21]. Most of these paracrine secretions include soluble factors and exosomes, which regulate the repair and regeneration processes at sites of damage by affecting cell proliferation, migration, and differentiation [22, 23]. Exosomes are a type of sphere- or dish-shaped extracellular vesicle whose diameter is between 30 and 150 nm. Exosomes are found in abundance in endosome-derived components, which are considered to play important roles in intercellular communication due to their ability to transfer “cargos” [24, 25]. By transporting “cargos” such as proteins, RNAs, DNAs, and lipids [26], exosomes regulate the eventual fate of recipient cells. Recently, several studies have shown that ADSC-derived exosomes can exert similar biological effects as ADSCs and enhance bone regeneration [27, 28].

However, how exosomes function in osteogenic differentiation of ADSCs still needs to be further explored. As exosome research techniques develop, researchers have discovered that exosomes can induce epigenetic changes and regulate the fate of receptor cells in the process of promoting bone tissue repair and regeneration, including promoting proliferation or inhibiting apoptosis. Proteins and RNAs play vital roles in such processes [29–31]. MicroRNAs (miRNAs) are endogenous non-protein-coding RNAs with a length of approximately 22 nt. They can act as quintessential posttranscriptional regulators and can regulate the expression of target genes, mainly through specific binding to the 3′ untranslated regions (UTRs) of target genes [32]. Targeted binding of miRNAs to mRNAs leads to recruitment of the targeted mRNAs to the RNA-induced silencing complex (RISC), which then leads to translation stoppage and degradation of mRNA [32, 33]. Through this process, miRNAs can inhibit protein expression of targeted mRNAs. In recent years, an increasing number of studies have demonstrated that miRNAs play important roles in bone formation [34, 35]. Additionally, it has been found that exosomes contain miRNAs [36], which can regulate the process of bone regeneration by targeting multiple genes in recipient cells [34]. Nevertheless, there is a lack of data regarding the global expression profiles of miRNAs in exosomes derived from undifferentiated and osteogenic-differentiated ADSCs.

In this study, we verified that only exosomes derived from osteogenically differentiated ADSCs can promote osteogenic differentiation of ADSCs. Moreover, we compared the expression profiles of miRNAs in exosomes derived from undifferentiated ADSCs with those from osteogenically differentiated ADSCs using microarray assays and performed bioinformatic analyses to further explore the biological functions of these differentially expressed miRNAs, which will lay the foundation for further study regarding ADSCs' osteogenic regulatory functions of exosomal miRNAs.

## 2. Material and Methods

The study was approved by the Ethics Committee of the First Hospital of China Medical University, Shenyang, China.

### 2.1. Isolation, Culture, and Characterization of ADSCs

#### 2.1.1. Isolation and Culture of ADSCs

Human adipose tissue was obtained from 6 female patients aged 26.67 ± 5.57 years undergoing liposuction and without metabolic disease, hepatitis, HIV, syphilis, and other systemic diseases, which might affect the ongoing study at the Department of Plastic Surgery, in the first hospital of China Medical University.

ADSCs were separated and expanded according to the methods previously reported [37, 38]. In brief, the obtained adipose tissue was added into 50 mL centrifuge tubes and washed with sterile phosphate-buffered saline (PBS (Gibco, USA)), then centrifuged at 1200 ×*g* for 5 min to remove red blood cells (RBCs). The above procedure was repeated 3–4 times before enzymatic digestion with 0.2% collagenase type I (Sigma, USA) at 37°C. Dulbecco's Modified Eagle's Medium/Nutrient F-12 Ham (DME F12 (HyClone, USA)), containing 10% fetal bovine serum (FBS (Gibco, USA)), was added to the digested lipoaspirates for 5 min in order to neutralize enzyme activity; this was followed by centrifugation at 1200 ×*g* for 5 min. The pellet was resuspended and filtered through a 100 *μ*m mesh filter to remove cellular debris. Finally, cells were plated in 75 cm^2^ culture flasks and incubated in culture medium (DME F12, 10% FBS, 1% Penicillin-Streptomycin Solution (Gibco, USA)) at 37°C in 5% CO_2_ with saturated humidity. The culture flasks were washed thoroughly with PBS to remove RBCs, and the medium was changed every two days. ADSCs were passaged until they were 90% confluent; 0.25% trypsin: 0.2% EDTA at a ratio of 1 : 3 was used to dissociate the cells. ADSCs at passage three were used for subsequent experiments.

#### 2.1.2. Characterization of ADSCs by Flow Cytometry

Cells at the third passage (P3) were digested with trypsin to form a single cell suspension solution, which was incubated with antibodies, including anti-CD34-PE, anti-CD31-APC, anti-CD45-PerCP-Cy5-5, anti-CD10-APC-Cy7 (BioLegend, USA), anti-CD13-PE, and anti-CD49d-PE-Cy7 (BD Biosciences, USA). All antibody incubations were performed at 37°C in the dark for 30 min. The cells were analyzed by flow cytometry (LSR II, BD Biosciences, USA) and Treestar FlowJo software.

#### 2.1.3. Multilineage Potential Assay of ADSCs

Third passage ADSCs were used to demonstrate their ability to differentiate into adipocytes, osteoblasts, and chondrocytes. Briefly, cells were seeded in a 24-well plate at a density of 5 × 10^3^ cells/cm^2^ in standard growth medium. According to the manufacturer's instructions, when the cells reached 90–100% confluence, the basal medium was replaced with complete OriCell™ osteogenic differentiation medium (Cyagen, USA) and complete MesenCult™ Adipogenic Differentiation Medium (Stem Cell Technologies, Canada) for aiding osteogenesis and adipogenesis of ADSCs. The medium was changed every 3 days for 2–4 weeks. Adipogenic and osteogenic differentiations were detected by oil red O (Cyagen, USA) and alizarin red S (Solarbio, China) staining, respectively.

To verify the chondrogenic differentiation ability of ADSCs, MesenCult™ Chondrogenic Differentiation Medium was added based on the manufacturer's instructions. Briefly, 5 × 10^5^ cells were resuspended in 0.5 mL of complete MesenCult™ Chondrogenic Differentiation Medium and added into 15 mL polypropylene tubes, followed by centrifugation at 300 ×*g* for 10 min. The caps of the tubes were loosened prior to incubation at 37°C under 5% CO_2_ for 3 days. Then, 0.5 mL complete MesenCult™ Chondrogenic Differentiation Medium was added to a final volume of 1 mL, and incubation was continued at 37°C under 5% CO_2_ for 3 days. The medium was changed every three days until day 28. Subsequently, the cartilage mass was fixed with formalin and embedded in paraffin. Finally, alcian blue (Solarbio, China) staining was performed to detect chondrogenic differentiation.

### 2.2. Extraction and Identification of ADSC-Derived Exosomes

When the cells reached 80%–90% confluence, we replaced the standard growth medium with serum-free medium and collected conditioned medium after incubating for an additional 24 h. Similarly, the cells in the experimental group were induced by treating with complete OriCell™ osteogenic differentiation medium (Cyagen, USA) for 14 days, and the conditioned medium was collected after 24 h of continuous culture with serum-free medium instead of the original medium. Exosomes were harvested by centrifugation and ultracentrifugation of the conditioned medium containing undifferentiated ADSCs (Exos_D0) and osteogenically differentiated ADSCs after 14 days (Exos_D14), respectively.

#### 2.2.1. Extraction of ADSC-Derived Exosomes

The conditioned medium was centrifuged at 300 ×*g* for 10 min at 4°C, and the supernatant was collected. Next, the supernatant was centrifuged at 2000 ×*g* for 20 min at 4°C, and the resulting supernatant was transferred to a new tube and centrifuged at 10000 ×*g* for 30 min in a 45TI rotor (Beckman, USA). Finally, the precipitates containing the exosomes and contaminating proteins were collected after centrifugation at 130000 ×*g* for 70 min and were resuspended in 50 mL PBS. The resuspension was recentrifuged under the same conditions as the previous step to obtain exosomes, which were resuspended in PBS and sterilized by filtration using a 0.45 *μ*m filter.

#### 2.2.2. Identification of ADSC-Derived Exosomes

The structure of exosomes was observed by transmission electron microscopy (TEM, (HITACHI, Japan)). Characteristic surface markers of exosomes such as TSG101, CD9, and calnexin were detected by western blotting, as described in Section 2.6.

#### 2.2.3. Uptake Assay of ADSC-Derived Exosomes *In Vitro*

To identify uptake of exosomes by ADSCs, exosomes were labeled with Dil (Beyotime Biotechnology, China) at a concentration of 10 *μ*M for 15 min, according to the manufacturer's instructions. Then, the exosomes were incubated with ADSCs for 6 h. Nuclei of ADSCs were stained with Hoechst33258 (Solarbio, China). Exosome uptake was observed by fluorescence microscopy (OLYMPUS, Japan).

### 2.3. Osteogenic Differentiation of ADSCs with Exosomes and Alizarin Red S (ARS) Assay

ADSCs (P3) were seeded in a 6-well plate at a density of 2 × 10^4^ cells/cm^2^ in standard growth medium until the cells reached 90% confluence. Subsequently, the standard growth medium was changed to complete OriCell™ osteogenic differentiation medium (Cyagen, USA) with the medium containing Exos_D0 or Exos_D14, whose concentration was 20 *μ*g/mL, compared to negative controls without simulation. The medium was changed every two days until day 21. According to the manufacturer's instructions, the cells were washed with PBS once or twice and fixed for 30 min with 2 mL of 4% neutral formaldehyde solution in each well. After the formaldehyde solution was aspirated away and wells washed with PBS twice, 1 mL of alizarin red S solution was added for 5 min. The plate was washed with PBS twice, followed by placement under a light microscope to observe the stained cells.

### 2.4. Alkaline Phosphatase (ALP) Activity Assay

The detected ADSCs were lysed by cell lysis buffer for Western and IP without inhibitors (Beyotime Biotechnology, China). Then, the supernatant was collected by centrifugation for semiquantitative analyses of ALP using an Alkaline Phosphatase Assay Kit (Beyotime Biotechnology, China) according to the manufacturer's instructions. As a common chromogenic substrate of phosphatase activity, paranitrophenol (p-nitrophenol) yields a yellow product under alkaline conditions. Optical density (OD) values were determined using a spectrophotometer (SpectraMax Plus384, Molecular Devices, USA) at 405 nm. Finally, we normalized ALP expression levels to the total cell protein content to obtain an absorbance index.

### 2.5. Isolation of Exosomal RNA

Exosomal RNA was isolated by the SeraMir Exosome RNA Purification kit (System Biosciences, USA), according to the manufacturer's instructions. The exosome RNA isolation protocol was mainly divided into three parts: exosome isolation and lysis, exoRNA purification, and exoRNA elution. For the isolation and lysis steps, culture medium was combined with ExoQuick-TC before thorough mixing by inversion three times. Then, the mixture was placed at 4°C for 6 h overnight and centrifuged at 11200 ×*g* for 2 min. After that, supernatant was removed and lysis buffer was added to the exosome pellet. After vortexing for 15 s, the mixture was placed at room temperature (25°C) for 5 min to allow complete lysis. For exoRNA purification, 100% ethanol was added before vortexing for 10 s. After the assembly of spin column and collection tubes, the mixture was transferred to spin columns and centrifuged at 11200 ×*g* for 1 min. The flow-through was discarded, and the spin column was placed back into a collection tube. Then, wash buffer was added before centrifugation at 11200 ×*g* for 1 min, and this step was repeated twice. Lastly, the flow-through was discarded and the mixture was centrifuged at 11200 ×*g* for 2 min to dry. For elution, the collection tube was discarded, and the spin column was assembled with a fresh, RNase-free 1.5 mL elution tube. Elution buffer was added directly to the spin column membrane and centrifuged at 300 ×*g* for 2 min and then 11200 ×*g* for 1 min to elute exoRNAs. After that, exosome RNA was recovered.

### 2.6. Quantitative Real-Time PCR (qPCR)

Extraction of cellular total RNAs and synthesis of cDNA were performed using TRIzol™ Reagent (Invitrogen, USA) and PrimeScript™ RT Master Mix (TAKARA, Japan), respectively, according to the manufacturer's instructions. Quantitative fluorescence detection was performed using TB Green™ Premix Ex Taq™ II (TAKARA, Japan) according to the manufacturer's instructions, under the PCR conditions of predenaturation at 95°C for 30 s, denaturation at 95°C for 5 s, and extension at 60°C for 30 s, for 40 cycles. The relative expression was calculated by the 2^-*ΔΔ*CT^ method, and each experiment was repeated 3 times. Sequences of PCR primers for Runt-related transcription factor 2 (*RUNX2*), alkaline phosphatase (*ALP*), glyceraldehyde-3-phosphate dehydrogenase (*GAPDH*), differentially expressed miRNAs (miR-130a-3p, miR-30b-5p, miR-34a-5p, miR-324-5p, miR-378f, and miR-513b-5p), and *U6* are given in Table 1. *GAPDH* was used for mRNA normalization. *U6* was used for miRNA normalization. We defined significant results according to *p* value threshold (<0.05) and FC (fold change) threshold (≥2).

### 2.7. Western Blotting

Proteins were separated by electrophoresis on 11% SDS-PAGE gels and transferred to PVDF membranes (Millipore, USA) and stained with Ponceau S staining solution (Beyotime Biotechnology, China) for 5-10 min. Membranes were then incubated with each primary antibody, including anti-ALP, anti-RUNX2, anti-TSG101, anti-CD9, and anti-calnexin (Abcam, USA, 1 : 1000 dilution) for 16 h, and the respective secondary antibody (Cell Signaling Technology, USA, 1 : 5000 dilution) after the membrane was blocked with 5% evaporated skimmed milk. After each incubation, the membrane was washed three times with TBST. Target bands were developed using an enhanced chemiluminescence (ECL) kit (Solarbio, China) according to the manufacturer's instructions. To quantify the results of western blots, we calculated mean Intden (integrated density) values using ImageJ 1.8.0 software. The relative intensity was measured by normalization using GAPDH.

### 2.8. Microarray Assays

Total RNAs of Exos_D0 and Exos_D14 were extracted from exosomes using TRIzol™ Reagent (Invitrogen, USA) according to the manufacturer's instructions. The quantity and quality of RNA were examined by NanoDrop 2000 and Agilent Bioanalyzer 2100. Expression profiles of miRNA were tested by GeneChip 4.0 (Affymetrix, USA) and verified using three parallel replicates.

### 2.9. Bioinformatics Analysis

Recognition of differentially expressed genes was performed using the limma package of the R program [39] with thresholds of *p* values < 0.05 and log_2_∣FC∣ ≥ 1(∣FC∣≥2). Differentially expressed genes in two datasets were selected for further analysis. In order to avoid biasing of results from different analysis platforms, we use DAVID Bioinformatics Resources 6.8 (https://david.ncifcrf.gov/) [40] for pathway analysis and gene ontology (GO) analyses, in which pathway analyses included Kyoto Encyclopedia of Genes and Genomes (KEGG) pathway analysis and Biocarta pathway analysis. GO analysis included biological process (BP), cellular component (CC), and molecular function (MF) categories. For the analysis results, we considered *p* values < 0.05 as significant. In addition, we selected 10 miRNAs with the most obvious differential expression to predict downstream target genes using three online analysis platforms: TargetScan (http://www.targetscan.org), microRNA.ORG (http://www.microrna.org/microrna/), and miRDB (http://mirdb.org/). We regarded the intersection of the three platforms as the ultimate target genes. In order to depict pathway and GO analysis results intuitively, we constructed enrichment analysis maps using R. The miRNA-Gene-Network was visually presented through Cytoscape 3.60.

### 2.10. Statistical Analysis

In this study, each experiment was checked by three parallel replicates to ensure the repeatability of the experiments. Statistical analysis was performed using SPSS 17.0 software and GraphPad Prism 7.0. For all data, *p* values < 0.05 were considered statistically significant.

## 3. Results

### 3.1. Identification of Human ADSCs

**Figure 1:**
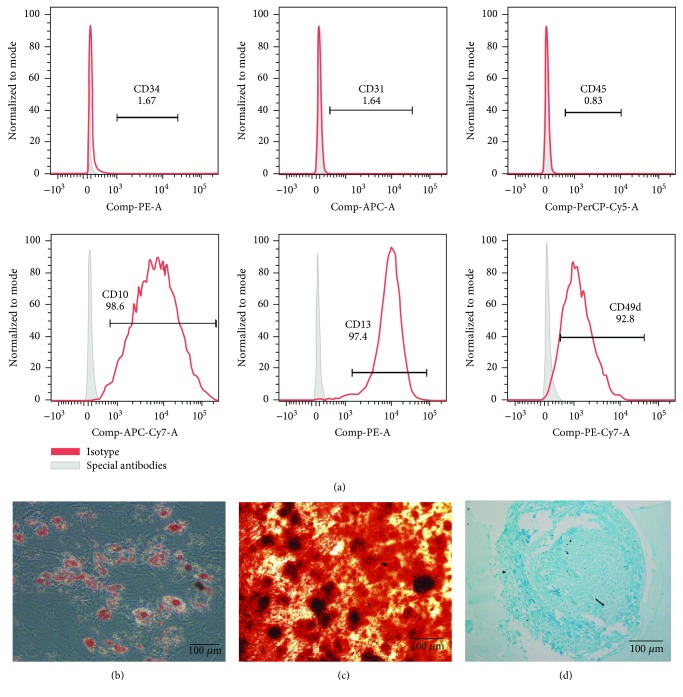
Characterization of ADSCs (P3). (a) The expression of the characteristic surface markers of ADSCs shown by flow cytometry. (b) Lipid droplets formed after 14 days of adipogenic induction, which is confirmed by oil red O staining. (c) Calcium nodules formed during osteogenic differentiation, which is identified by ARS after 28 days of osteogenic induction. (d) The chondrocytes stained by alcian blue after 28 days of chondrogenic induction. Notes: ADSCs: human adipose-derived stem cells; ARS: alizarin red S. Scale bar: 100 *μ*m.

ADSCs that we used in this study were obtained by a method involving collagenase digestion and adherent cell culture. We detected characteristic ADSC surface markers using flow cytometry and obtained the following results: CD10, CD13, and CD49d expression was positive, while the expression of CD34, CD31, and CD45 was negative, as shown in Figure 1(a). Importantly, ADSCs can differentiate into adipocytes, osteoblasts, and chondrocytes (Figures 1(b)–1(d)), confirming multilineage potential, in line with the recognized standard for identification of ADSCs [9, 41].

### 3.2. Exosomes Derived Only from Osteogenically Differentiated ADSCs Can Promote Osteogenic Differentiation

#### 3.2.1. Identification of ADSC-Derived Exosomes

**Figure 2:**
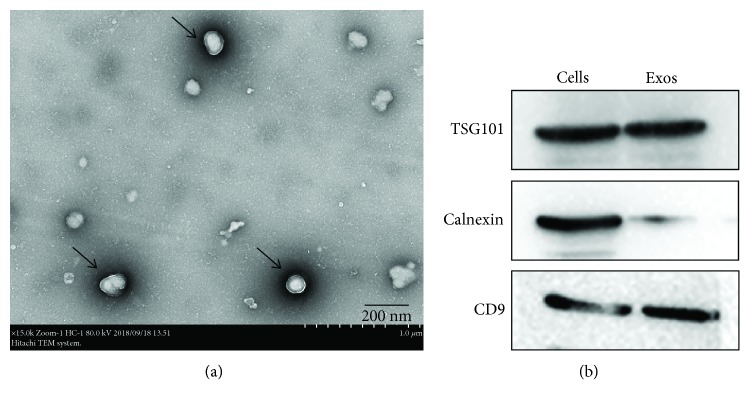
Identification of ADSCs-derived exosomes from the conditioned medium. (a) The image of TEM of ADSC-derived exosomes, scale bar: 200 nm. (b) Western blot analysis of the exosome-associated protein TSG101 and CD9 was expressed while endoplasmic reticulum proteins calnexin was hardly expressed. Notes: TEM: transmission electron microscopy.

ADSC-derived exosomes were isolated by centrifugation and ultracentrifugation from conditioned medium. As shown in Figure 2(a), TEM revealed that vesicles with particle sizes between 30 nm and 150 nm exhibited spherical morphology, proving the presence of exosomes. In addition, the western blot results showed that the exosome-associated proteins TSG101 and CD9 were expressed while the endoplasmic reticulum protein calnexin was hardly expressed in exosomes (Figure 2(b)). The above results demonstrate that we successfully extracted ADSC-derived exosomes.

#### 3.2.2. Exosome Uptake by ADSCs

**Figure 3:**
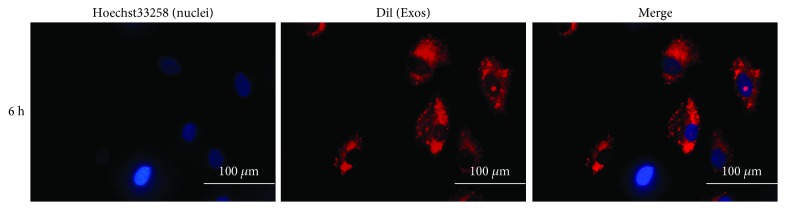
Internalization of exosomes by ADSCs. ADSCs can ingest a large number of exosomes at only 6 h postincubation. The results shown in Figure S1 further confirmed that exosomes can be internalized by ADSCs. Exosomes were labeled with Dil (red), and the nuclei were stained with Hoechst33258 (blue). Scale bar: 100 *μ*m.

To explore whether ADSCs can internalize exosomes, we incubated Dil-labeled exosomes with ADSCs for 6 h. As shown in Figure 3, exosomes labeled with Dil (red dots) were gradually internalized by ADSCs. Many exosomes were observed in the cytoplasm of their homotypic cells—ADSCs at only 6 h postincubation by fluorescence microscopy.

#### 3.2.3. Exosomes Derived from Osteogenically Differentiated and Undifferentiated ADSCs Promote Osteogenic Differentiation of ADSCs

To investigate differences in exosome function, exosomes derived from undifferentiated ADSCs (Exos_D0) and osteogenically differentiated ADSCs at 14 days (Exos_D14) were extracted. Exos_D0 and Exos_D14 were used as stimuli to treat third passage ADSCs without other osteogenic interference factors. In order to establish more accurate results, we set-up 4 comparative groups: a negative control (NC) group, a positive control (PC) group, an Exos_D0 group, and an Exos_D14 group. ADSCs in the PC group were induced with osteogenic differentiation medium, while the NC group remained untreated.

**Figure 4:**
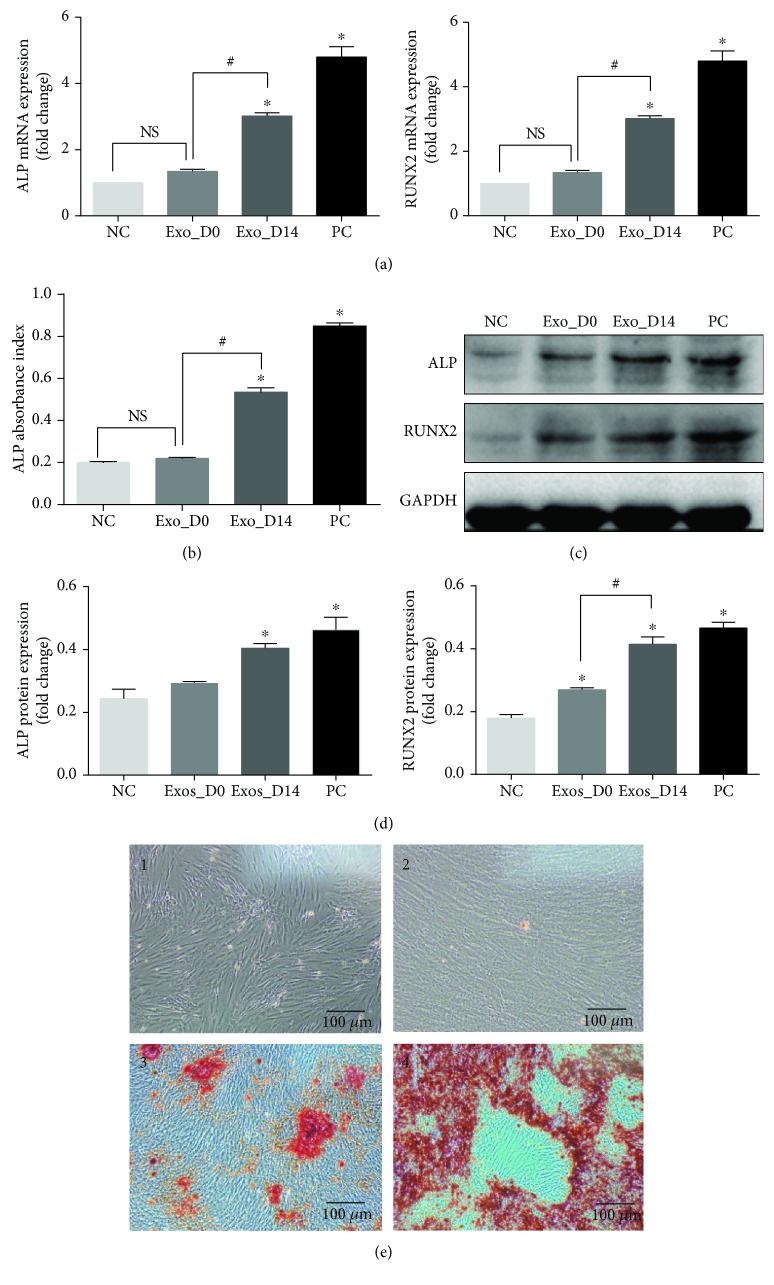
The Potential of exosomes' ability to promote osteogenic differentiation of ADSCs. (a) qPCR analysis expression of osteogenesis-related genes (ALP, RUNX2) in NC, Exos_D0, Exos_D14, and PC on 7th day. (b) Semiquantitative analyses of ALP activity on 7th day. (c) Western blot analyses of the protein expression of ALP and RUNX2 on 14th day. (d) Relative intensity analyses of western blot results of ALP and RUNX2. (e) ARS staining on 21st day (1 represents NC, 2 represents Exos_D0, 3 represents Exos_D14, and 4 represents PC), scale bar: 100 *μ*m. Notes: ^∗^represents significant differences between NC and other groups; ^#^represents significant differences between Exos_D14 and Exos_D0; NS represents no significant differences. ^∗^ *p* < 0.05; ^#^ *p* < 0.05.

qPCR and ALP activity assays were performed 7 days after treatment. Compared with NC and Exos_D0, the expression of osteogenesis-related genes such as *ALP* and *RUNX2* was significantly increased (FC > 2, *p* < 0.05) in the Exos_D14 and PC groups. Interestingly, there were no significant differences between the NC and Exos_D0 groups, but the difference between the Exos_D0 and Exos_D14 groups was obvious (Figure 4(a)). ALP semiquantitative analyses also showed similar results to those of qPCR. ALP activity was dramatically higher in the Exos_D14 and PC groups. In comparisons between NC and Exos_D0, Exos_D0 and Exos_D14 groups, the results exhibited no significant differences and notable differences (Figure 4(b)). Additionally, western blot analysis was corroborated by qPCR results in that protein expression of ALP, and RUNX2 was remarkably elevated in the Exos_D14 and PC groups, compared with NC and Exos_D0, as shown in Figures 4(c) and 4(d).

After 21 days of treatment, alizarin red staining revealed that there were significantly more calcium nodules formed in the Exos_D14 and PC groups (Figure 4(e)). These results confirmed that Exos_D14 rather than Exos_D0 can effectively promote osteogenic differentiation of ADSCs.

### 3.3. Distinct Levels of miRNA Expression in Exosomes Derived from Osteogenically Differentiated ADSCs and Undifferentiated ADSCs

**Figure 5:**
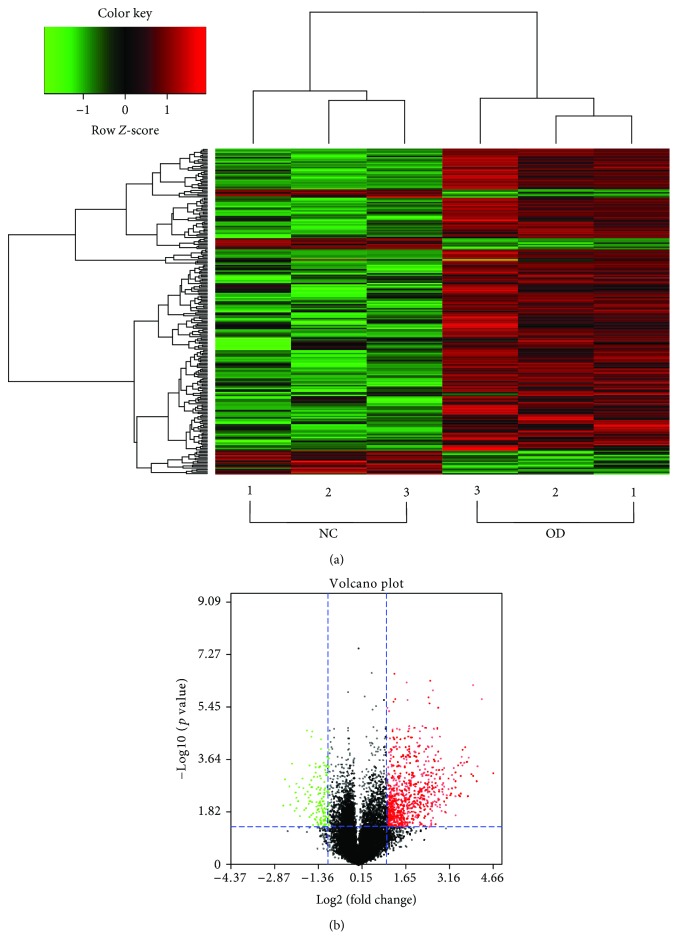
The miRNA differential expression profiles in exosomes. (a) The heat map of distinct miRNAs in exosomes derived from undifferentiated and osteogenically differentiated ADSCs based on microarray. (b) The volcano plot of miRNAs in exosomes derived from undifferentiated and osteogenically differentiated ADSCs. The red dots represent upregulation, and the green dots represent downregulation of expression with statistical significance (fold change ≥2, *p* value < 0.05). Notes: NC: normal control (exosomes derived from undifferentiated ADSCs); OD: osteogenic differentiation (exosomes derived from osteogenically differentiated ADSCs).

In order to understand the underlying mechanisms of how exosomes promoted osteogenic differentiation of ADSCs, the miRNA expression profiles of exosomes derived from undifferentiated and osteogenically differentiated ADSCs were analyzed by microarray. We detected a total of 2170 mature miRNAs and 1868 pre-miRNAs expressed in ADSC-derived exosomes in which hierarchical clustering showed that miRNA expression profiles in the same group were consistent, while the expression profiles between the two groups were distinct. As shown in Figure 5(a), the abscissa represents sample clustering (the first three columns represent exosomes derived from undifferentiated ADSCs, and the last three columns represent exosomes derived from osteogenically differentiated ADSCs) and the ordinate represents gene clustering. The deeper the red, the more obvious the upregulation of the gene, and the deeper the green, the more obvious the downregulation of the gene.

Compared with exosomes derived from undifferentiated ADSCs, 201 miRNAs were upregulated and 33 miRNAs were downregulated in exosomes derived from osteogenically differentiated ADSCs (FC ≥ 2, *p* value < 0.05), as represented by a volcano plot (Figure 5(b)).

### 3.4. Exosomal miRNAs Affect Osteogenic Differentiation via a Series of Biological Processes

Exosomal miRNAs can affect the osteogenic differentiation process through a series of biological processes in which they first target related genes such that they can regulate axon guidance, MAPK signaling, and Wnt signaling and modify gene expression, cell metabolism, and other biological functions.

#### 3.4.1. Pathway Analysis and Functional Analysis of Exosomal miRNAs

**Figure 6:**
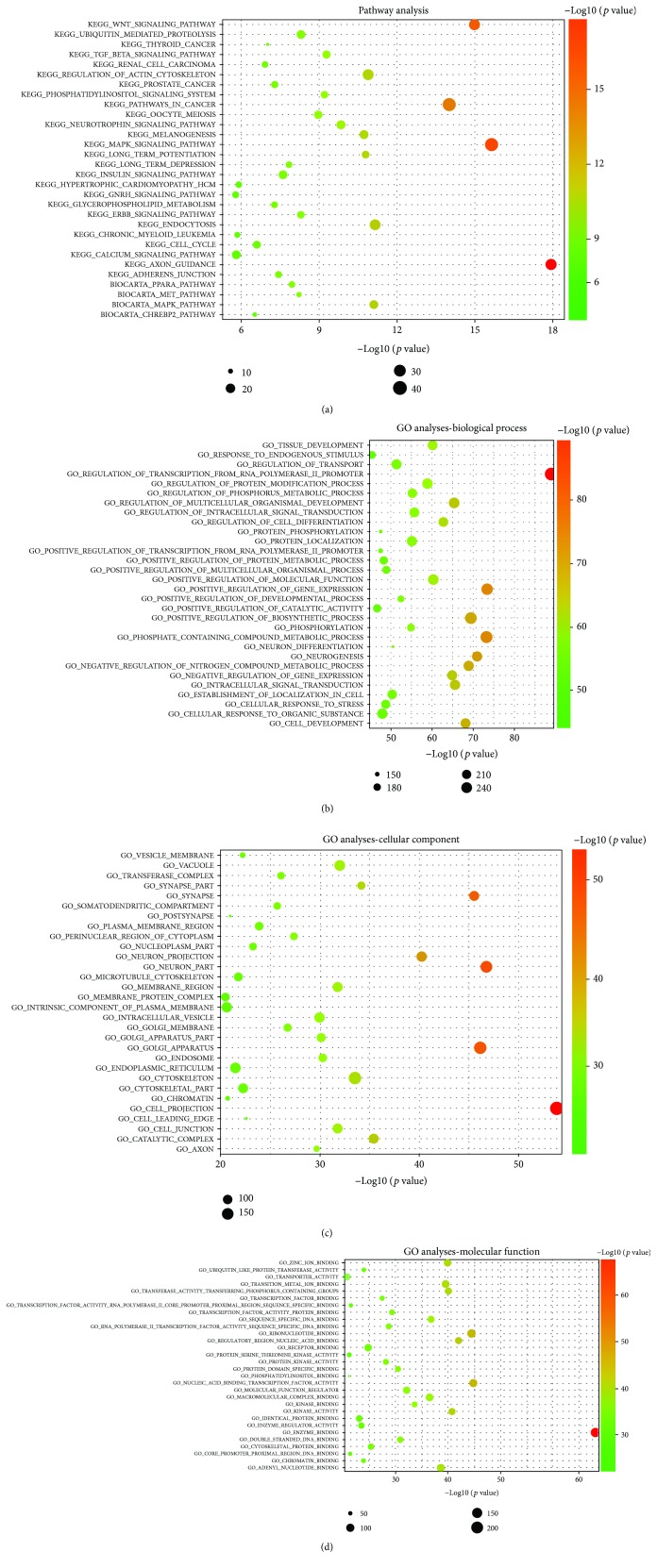
Pathway analysis and GO analyses. (a) Enrichment map of KEGG pathway analysis and Biocarta pathway analysis. (b) Enrichment map of GO analyses—biological process analysis. (c) Enrichment map of GO analyses—cellular component analysis. (d) Enrichment map of GO analyses—molecular function. Notes: KEGG: Kyoto Encyclopedia of Genes and Genomes pathway analysis; GO: gene ontology.

To determine which signaling pathways were changed during osteogenic differentiation of ADSCs regulated by exogenous microRNAs, KEGG pathway analysis and Biocarta pathway analysis were performed. The results revealed that the target genes of the differentially expressed miRNAs are principally related to processes such as axon guidance (*p* value = 1.16*E*-18), MAPK signaling (*p* value = 2.31*E*-16), Wnt signaling (*p* value = 1.03*E*-15), endocytosis (*p* value = 7.1*E*-12), regulation of actin cytoskeleton (*p* value = 1.31*E*-11), and the TGF-*β* signaling pathway (*p* value = 5.26*E*-10) (Figure 6(a)). It suggested that these biologic pathways were involved in osteogenic differentiation of ADSCs.

To explore the potential biological functions of differentially expressed miRNAs, we performed GO enrichment analyses, including BP, CC, and MF by DAVID. According to statistically significant GO analysis results (*p* values < 0.05), we found that functions such as enzyme binding (*p* value = 7.7*E*-69), cell projection (*p* value = 1.29*E*-54), transcription factor activity (*p* value = 1.53*E*-45), regulation of gene expression (*p* value = 4.52*E*-74), and cell metabolism (*p* value = 5.92*E*-74) are mainly affected by differentially expressed miRNAs (Figures 6(b)–6(d)). Interestingly, these functions were closely related to osteogenic differentiation of ADSCs.

#### 3.4.2. miRNA-mRNA Network Analysis

**Figure 7:**
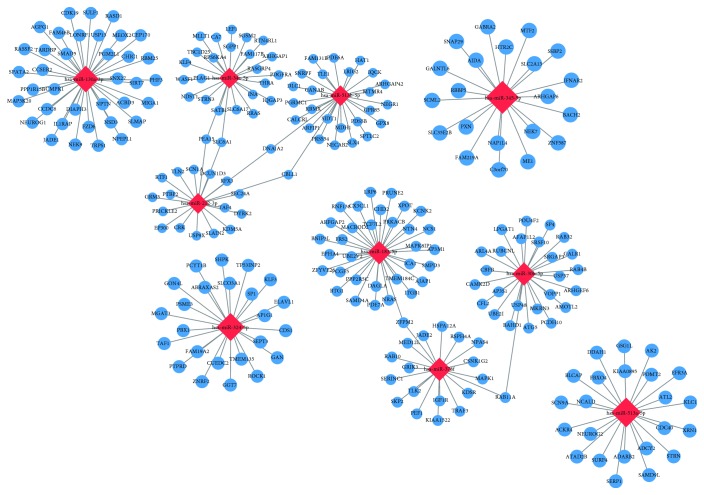
miRNA-mRNA network. The relationship between differentially expressed miRNAs and predicted downstream mRNA is clearly shown in the miRNA-mRNA network, which reveals that one miRNA may recognize multiple target mRNAs simultaneously, and one gene may also be regulated by multiple miRNAs. Notes: miRNA: microRNA; mRNA: messenger RNA.

miRNAs can target single or multiple genes involved in the same or different signaling pathways in order to regulate the process of bone regeneration [30]. To explore the link between differentially expressed miRNAs and associated protein-encoding mRNAs, we chose 10 miRNAs (miR-130a-3p, miR-30b-5p, miR-34a-5p, miR-183-5p, miR-212-3p, miR-324-5p, miR-345-5p, miR-378f, miR-513a-5p, and miR-513b-5p) with the most obvious differential expression to predict downstream target genes using TargetScan, microRNA.ORG, miRDB, and constructed miRNA-mRNA networks using Cytoscape. As shown in Figure 7, we discovered that one miRNA may recognize multiple target mRNAs simultaneously, and one gene may also be regulated by multiple miRNAs. More importantly, a great quantity of miRNAs was predicted to be involved in a variety of pathways that promote osteogenic differentiation. For instance, mir-130a-3p was a miRNA with the highest differential expression and which was predicted to have a high probability of binding to *SIRT7*. Previous research has found that knockdown of SIRT7 enhances osteogenic differentiation of bone marrow mesenchymal stem cells (BMSCs) [42].

#### 3.4.3. qPCR Validation of miRNA Expression

**Figure 8:**
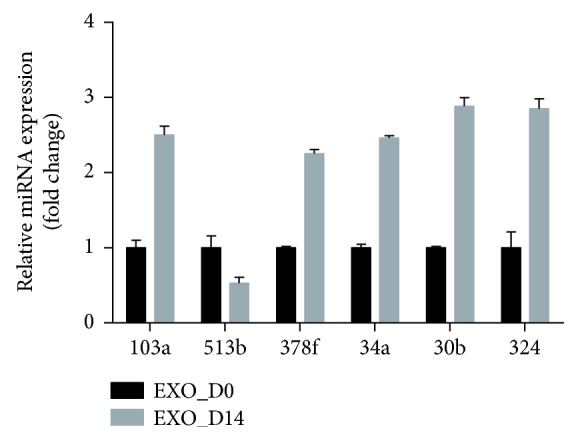
Comparison of the expression of chosen miRNA between Exos_D0 and Exos_D14 and qPCR results. For qPCR analyses, miRNA expression was normalized to U6 expression. Note: ^∗^represents significant differences between Exos_D0 and Exos_D14. ^∗^ *p* < 0.05.

The 6 miRNAs (miR-130a-3p, miR-513b-5p, miR-30b-5p, miR-34a-5p, miR-324-5p, and miR-378f) with the most obvious differential expression were chosen to validate the results of microarray analyses using qPCR. In agreement with the preliminary conclusions obtained by microarray, compared with exosomes derived from undifferentiated ADSCs, the expression of 5 miRNAs (miR-130a-3p, miR-30b-5p, miR-34a-5p, miR-324-5p, and miR-378f) in exosomes derived from osteogenically differentiated ADSCs was significantly increased (FC > 2, *p* < 0.05) while the expression of miR-513b-5p was decreased (Figure 8). qPCR results confirmed the validity of differentially expressed miRNAs identified by microarray, which revealed that these miRNAs have functions in regulating ADSC osteogenesis.

## 4. Discussion

A major issue in the field of bone regeneration is inducing differentiation of stem cells into osteoblasts. In addition to osteogenic differentiation medium, genetic modification, and growth factors, MSC-derived exosomes have also drawn much attention in recent years because of their ability to induce osteogenic differentiation of stem cells [28, 43, 44]. However, ADSC-derived exosomes have rarely been examined in the field of bone regeneration. In our current study, we explored the differences between the effects of exosomes derived from osteogenically differentiated ADSCs and undifferentiated ADSCs separately on osteogenic differentiation of ADSCs *in vitro*. Our results indicated that osteogenically differentiated ADSC-derived exosomes can promote osteogenic differentiation of ADSCs, whereas undifferentiated ADSC-derived exosomes cannot (Figure 4). This finding provides evidence that ADSC-derived exosomes can be an ideal inducing factor with excellent osteogenic efficacy, safety, and widespread availability in bone regeneration and clinical applications. Li et al. have proven that the addition of ADSC-derived exosomes to osteogenic differentiation medium can promote osteogenic differentiation of bone marrow MSCs *in vitro* [28]. To our knowledge, in the absence of factors intervening in osteogenic differentiation, for the first time, we have demonstrated excellent osteogenic activity of osteogenically differentiated ADSC-derived exosomes. In addition, we found that it took only 6 h for ADSCs to ingest a large number of ADSC-derived exosomes (Figure 3), compared to 48 h for bone marrow-derived stem cells (BMSCs) [28]. We speculated that perhaps this may be because ADSC-derived exosomes are more easily ingested by homotypic cells, namely, ADSCs. This suggests that the combination of ADSC-derived exosomes and ADSCs in bone tissue engineering can reduce the loss of exosomes, which is due to a longer uptake time, thus promoting bone regeneration more efficiently.

Understanding the precise molecular mechanisms of osteogenesis is of immense importance for promoting the osteogenic efficacy and clinical application of stem cells in bone regeneration. To date, most research has focused on the study of epigenetic and transcriptional factors involving stem cells themselves [45–47], whereas few studies have focused on the effects of exosome “cargo” on the osteogenic differentiation of stem cells. It has been noted that exosomes can regulate corresponding biological processes by affecting related pathways in receptor cells. For example, Yue et al. showed that huc-MSC-derived exosomes promote cutaneous wound healing by influencing Wnt4 signaling [48]. Zhang et al. demonstrated that exosomes can enhance bone regeneration by activating the PI3K/Akt signaling pathway [49]. Interestingly, microRNAs can regulate the expression of mRNA by binding specifically to the 3′UTR of target genes, thus affecting the corresponding signaling pathways [32]. Based on previous studies, we have learned that there are proteins, DNAs, RNAs, lipids, and other biomolecules in exosomes, among which proteins and RNAs play important roles [25, 28]. miRNAs, an important class of RNA, are involved in a series of crucial processes, including cell growth, cell proliferation, differentiation, apoptosis, and cell death. Numerous reviews have mentioned that miRNAs play a critical role in bone biology [50–52]. Moreover, there are no reports concerning the genome-wide expression and function of miRNAs in ADSC-derived exosomes. We analyzed differences in the expression of miRNAs between undifferentiated ADSC-derived exosomes and osteogenically differentiated ADSC-derived exosomes by microarray assays.

Dysregulation of exosome-derived miRNA expression has been reported in schizophrenia and localized breast cancer [53, 54]. These studies suggested that the expression of miRNAs in exosomes changed significantly under different conditions. In this study, we detected 2170 mature miRNAs and 1868 pre-miRNAs in ADSC-derived exosomes. Compared with undifferentiated ADSC-derived exosomes, there were 234 significantly differentially expressed miRNAs (201 upregulated and 33 downregulated) in osteogenically differentiated ADSC-derived exosomes. A heat map of distinct miRNAs in exosomes indicates that miRNAs may play an important regulatory role in the process of osteogenic differentiation of ADSCs, promoted by exosomes.

miRNAs act through base pairing with complementary sequences within mRNAs and silence expression of corresponding genes, thus interfering with signaling pathways [55]. Furthermore, 10 differentially expressed miRNAs showing a high fold change were selected to predict their target genes and to perform pathway analysis. We found that one miRNA can target multiple mRNAs and that one mRNA can be regulated by one or more miRNAs. This conclusion is consistent with previous reports [56]. In our study, a majority of predicted miRNAs involved in signaling pathways were related to osteogenic differentiation, including the MAPK signaling pathway, Wnt signaling pathway, and TGF-*β* signaling pathway, among others (Figure 6(a)). For example, *SIRT7* is predicted to be a highly likely target of miR-130a-3p, with the highest fold change. We also confirmed that the expression of miR-130a-3p was significantly increased in Exo_D14, compared with Exos_D0 (Figure 8). Previous studies have demonstrated that SIRT7 inhibits osteogenic differentiation of BMSCs by antagonizing the Wnt signaling pathway, while mir-130a-3p promotes it [42, 57], which is consistent with our prediction. It is a fact that the Wnt signaling pathway is a highly conserved pathway involved in the regulation of cell growth, differentiation, survival, and apoptosis and plays a key role in the self-renewal and maintenance of stem cells [58]. To date, many studies have confirmed the important regulatory role of the Wnt signaling pathway in the process of osteogenic differentiation of stem cells [59, 60]. Consequently, the mir-130a-3p/SIRT7/Wnt axis may be a novel molecular mechanism regulating osteogenic differentiation of ADSCs. To further clarify the biological function of target genes of differentially expressed microRNAs, GO analyses, including BP, CC, and MF, were carried out. The results of the GO analyses revealed that the affected target genes are mainly involved in enzyme binding, cell projection, transcription factor activity, regulation of gene expression, and cell metabolism (Figures 6(b)–6(d)). It is clear that certain differentially expressed miRNAs in exosomes are closely related to cellular and molecular responses in the osteogenic differentiation process of ADSCs.

Our results indicated that exosomal miRNAs may play a vital role in enhancing bone regeneration. Exosomes can be ingested more rapidly by homotypic cells, as opposed to other cells. We believe that the combination of ADSC-derived exosomes and ADSCs will serve as excellent “inducing factors” and “seed cells” during the creation of tissue engineered bone—a development that would aid the repair of clinical bone defects. Moreover, the detailed mechanisms of how ADSC-derived exosomes enhance osteogenic differentiation of ADSCs require further exploration, but these comprehensive analytical experiments will build a rich information base for understanding the mechanisms underlying exosome-mediated promotion of osteogenic differentiation of ADSCs.

We should indicate that there are some limitations to this study. We only compared exosomes derived from osteogenically differentiated ADSCs and undifferentiated ADSCs. This may lead us to lose information regarding the dynamic changes in exosomal miRNAs throughout the osteogenic differentiation process. In addition, the specific molecular mechanisms involving exosomes remain elusive. Therefore, these issues should be explored in future studies.

## 5. Conclusion

In summary, to the best of our knowledge, we demonstrated for the first time that osteogenically differentiated ADSC-derived exosomes can promote osteogenic differentiation of ADSCs. Importantly, we successfully identified miRNAs of exosomes and performed functional analyses involving coregulated networks. These will serve as a new foundation for basic research in bone regeneration and clinical bone regeneration therapy.

## Figures and Tables

**Table 1:** List of gene primers.

Gene	Forward sequence	Reverse sequence
ALP	CAACGAGGTCATCTCCGTGATG	TACCAGTTGCGGTTCACCGTGT
RUNX2	CCCAGTATGAGAGTAGGTGTCC	GGGTAAGACTGGTCATAGGACC
GAPDH	GTCTCCTCTGACTTCAACAGCG	ACCACCCTGTTGCTGTAGCCAA
U6	CTCGCTTCGGCAGCACA	AACGCTTCACGAATTTGCGT
miR-130a-3p	GATGCTCTCAGTGCAATGTTA	
miR-34a-5p	AGCCGCTGGCAGTGTCTTA	
miR-30b-5p	GCTGCCGTTGTAAACATCCTAC	
miR-324-5p	CAGCCTAATCGCATCCCCTA	
miR-513b-5p	GCCGCTTCACAAGGAGGT	
miR-378f	GCTGGGACTGGACTTGGA	

## Data Availability

The data used to support the findings of this study are available from the corresponding author upon request.

## Supplementary Materials

Supplementary Materials (a) The result of real-time imaging by fluorescence confocal microscopy showed the process that ADSCs gradually internalized homologous exosomes labeled with Dil (the red dots) from 0 minute to 360 minute. (b) 9 time points (1 min, 30 min, 60 min, 90 min, 120 min, 180 min, 240 min, 300 min, and 360 min) in the same field of view were chosen to acquire images.
