# Supplementary material for: Promoting Osteogenic Differentiation of Human Adipose-Derived Stem Cells by Altering the Expression of Exosomal miRNA
Source: Stem Cells Int. 2019 Jul 1;2019:1351860. doi: 10.1155/2019/1351860 (PMC6636464; doi:10.1155/2019/1351860)
Supplement: Supplementary Materials — (a) The result of real-time imaging by fluorescence confocal microscopy showed the process that ADSCs gradually internalized homologous exosomes labeled with Dil (the red dots) from 0 minute to 360 minute. (b) 9 time points (1 min, 30 min, 60 min, 90 min, 120 min, 180 min, 240 min, 300 min, and 360 min) in the same field of view were chosen to acquire images. [file 1351860.f1.zip › 1351860.f2.pdf]

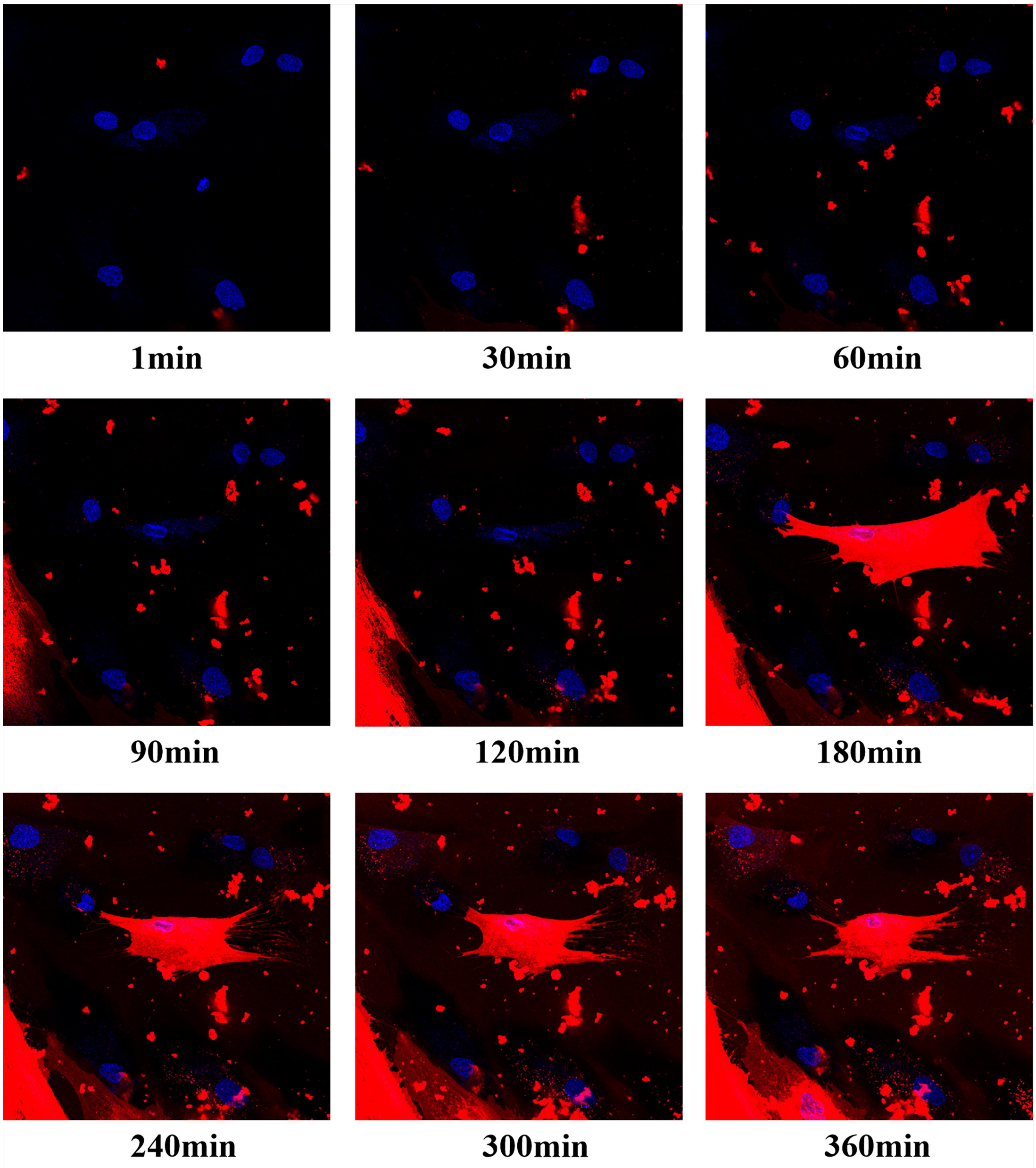

(b)

Fig S1. Internalization of exosomes by ADSCs. (a)The result of real-time imaging by fluorescence confocal microscopy showed the process that ADSCs gradually internalized homologous exosomes labeled with Dil (the red dots) from 0 minute to 360 minute. (b) 9 time points (1min, 30min, 60min, 90min, 120min, 180min, 240min, 300min, 360min) in the same field of view was chosen to acquire images. The results further confirmd the conclusion.
